# Recombination-mediated dissemination of Methicillin-resistant *S. aureus* clonal complex 1 in the Egyptian health care settings

**DOI:** 10.1186/s12941-023-00659-y

**Published:** 2023-12-14

**Authors:** Salma W. Elsayed, Reem A. Elghaish, Eman Badr, Shaimaa F. Mouftah, Nehal A. Saif, Iman S. Naga, Ahmed H. Shata, Ben Pascoe, Samuel K. Sheppard, Mohamed Elhadidy

**Affiliations:** 1 Center for Genomics, Helmy Institute for Medical Sciences, Zewail City of Science and Technology, Giza, Egypt; 2 Biomedical Sciences Program, University of Science and Technology, Zewail City of Science and Technology, Giza, Egypt; 3 Department of Microbiology & Immunology, Faculty of Pharmacy, Ain Shams University, Cairo, Egypt; 4 The University of Texas MD Anderson Cancer Center UTHealth Graduate School of Biomedical Sciences, Houston, TX USA; 5 Faculty of Computers and Artificial Intelligence, Cairo University, Giza, Egypt; 6 Department of Microbiology, Medical Research Institute, Alexandria University, Alexandria, Egypt; 7 Centre for Genomic Pathogen Surveillance, Big Data Institute, University of Oxford, Oxford, UK; 8 Ineos Oxford Institute, Department of Biology, University of Oxford, Oxford, UK; 9 Department of Bacteriology, Mycology and Immunology, Faculty of Veterinary Medicine, Mansoura University, Mansoura, Egypt

**Keywords:** Methicillin-resistant *Staphylococcus aureus* (MRSA), Hospital settings, Antimicrobial resistance, Virulence, Recombination, Linezolid resistance

## Abstract

**Background:**

Methicillin-resistant *Staphylococcus aureus* (MRSA) is a rapidly evolving pathogen that is frequently associated with outbreaks and sustained epidemics. This study investigated the population structure, resistome, virulome, and the correlation between antimicrobial resistance determinants with phenotypic resistance profiles of 36 representative hospital-acquired MRSA isolates recovered from hospital settings in Egypt.

**Results:**

The community-acquired MRSA lineage, clonal complex 1 (CC1) was the most frequently detected clone, followed by three other globally disseminated clones, CC121, CC8, and CC22. Most isolates carried SCC*mec* type V and more than half of isolates demonstrated multi-drug resistant phenotypes. Resistance to linezolid, a last resort antibiotic for treating multidrug resistant MRSA, was observed in 11.11% of the isolates belonging to different genetic backgrounds. Virulome analysis indicated that most isolates harboured a large pool of virulence factors and toxins. Genes encoding aureolysin, gamma hemolysins, and serine proteases were the most frequently detected virulence encoding genes. CC1 was observed to have a high pool of AMR resistance determinants including *cfr*, *qac*A, and *qac*B genes, which are involved in linezolid and quaternary ammonium compounds resistance, as well as high content of virulence-related genes, including both of the PVL toxin genes. Molecular clock analysis revealed that CC1 had the greatest frequency of recombination (compared to mutation) among the four major clones, supporting the role of horizontal gene transfer in modulating AMR and hypervirulence in this clone.

**Conclusions:**

This pilot study provided evidence on the dissemination success of CA-MRSA clone CC1 among Egyptian hospitals. Co-detection of multiple AMR and virulence genes in this lineage pose a broad public health risk, with implications for successful treatment. The results of this study, together with other surveillance studies in Egypt, should be used to develop strategies for controlling MRSA infections in Egyptian health-care settings.

**Supplementary Information:**

The online version contains supplementary material available at 10.1186/s12941-023-00659-y.

## Background

*Staphylococcus aureus* (*S. aureus*) is an opportunistic and a commensal bacterial pathogen that often colonizes skin and mucus membranes asymptomatically [24]. It can cause a plethora of diseases ranging from mild illnesses to severe life-threatening conditions [35]. Additionally, *S. aureus* is equipped with a high number of virulence factors and toxins, enabling the bacteria to combat host immunity and impact disease outcomes. Subsequently, the ability of *S. aureus* to acquire resistance to a wide range of clinically used antibiotics complicates its treatment [32].

*S. aureus* belongs to the notorious ESKAPE pathogens (*Enterococcus faecium, Staphylococcus aureus, Klebsiella pneumoniae, Acinetobacter baumannii, Pseudomonas aeruginosa, and Enterobacter species*) that are known for their growing multidrug resistance [39]. These pathogens represent a major cause of nosocomial infections and demand urgent development of new antibiotics for their control [39]. Methicillin-resistant *S. aureus* (MRSA) was first identified in the 1960s, shortly after the clinical introduction of methicillin [57]. The acquisition and chromosomal integration of *mecA* gene, located in the mobile genetic element (MGE) designated staphylococcal cassette chromosome mec (SCC*mec*), which encodes for an altered penicillin-binding protein (PBP), is the genetic mechanism underlying the decreased affinity to methicillin and most semisynthetic beta-lactam antibiotics in MRSA [57]. Furthermore, MRSA have also developed co-resistance to other classes of antibiotics (such as aminoglycosides, macrolides, fluoroquinolones, and clindamycin), limiting treatment options. Thus, MRSA strains, together with other possible resistance mechanisms, might be considered as the first class multidrug resistant (MDR) pathogens [23]. Hence, the escalation in antimicrobial resistance (AMR) among MRSA represents a serious public health threat. Furthermore, MRSA-mediated infections are challenging to control, and associated with critical complications and prolonged hospitalization, making them logistically and economically burdening [58].

The epidemiology of MRSA infections has changed in the past two decades. At first, MRSA was confined to health care settings, with infections reported exclusively from immunocompromised patients and personnel exposed to hospital environments [32]. Shortly afterwards, community-acquired strains emerged and became a predominant cause of infections in otherwise healthy individuals. Community-acquired MRSA (CA-MRSA) and hospital-acquired MRSA (HA-MRSA) had distinct genotypic and phenotypic characteristics that allowed them to thrive in their ecological niches. For instance, HA-MRSA is more resistant to a broader range of antibiotics [42]. CA-MRSA, on the other hand, is more sensitive to antibiotics but rather more virulent [22, 40]. Dissemination of both AMR and virulence factors is mediated by horizontal gene transfer (HGT) of mobile genetic elements (MGEs) and by mutations [6, 27]. Other molecular features like SCC*mec* type and the presence of Panton-Valentine leukocidin (PVL) toxin genes are classically used to differentiate HA-MRSA from CA-MRSA [40]. However, a clear-cut between these two types is gradually diminishing due to their increasing shared molecular and epidemiological characteristics [31, 32]. CA-MRSA strains are increasingly replacing traditional HA-MRSA ones in health care settings blurring their classical definition [14, 15]. Besides colonizing humans, MRSA had successfully gained a foothold in livestock and companion animals, expanding its host range and creating additional reservoirs [41, 43]. MRSA epidemiology and population structure vary with geographical locations [4]. Specific high risk MRSA lineages have spread around the world and become epidemic in MRSA-mediated infections, with different lineages dominating in different parts of the world, at different times [32]. Continuous surveillance of MRSA isolates obtained from local healthcare units is therefore pivotal in assessing existing infection control programs and optimizing treatment options, especially in endemic regions. Although MRSA prevalence in Egypt is one of the highest among African countries and Mediterranean regions, genomic epidemiology of circulating clones remains scarce [5].

This study aims to investigate the clonal distribution and recent evolving trends of MRSA isolates recovered from health care settings in Egypt and to identify their resistance determinants and virulence markers. Overall, our results demonstrate the usefulness of genomic surveillance of MRSA strains in Egypt and reveal genomic adaptations of diverse MRSA clones, particularly CC1 triggered by the improper use of antibiotics at the hospital environment.

## Materials and methods

### Bacterial cultures and antibiotic resistance testing

A total of 36 representative non-duplicate MRSA isolates were obtained from different internal medicine wards in tertiary care hospitals in Alexandria, Egypt during the period from 2020 to 2021. These isolates were classified as HA-MRSA according to the standard epidemiological criteria of the US Centers for Disease Control and Prevention (CDC) [7]. Briefly, CA-MRSA infection is defined in individuals within 48 h after hospital admission, who had no history of hospitalization or surgery, and no previous positive culture for MRSA. HA-MRSA on the other hand is detected by a positive culture taken more than 48 h after hospitalization. Isolates were obtained from various sources including blood cultures, ulcer swabs, wound swabs, sputum, urine, breast abscesses, and axillary abscesses. MRSA confirmation was preformed using Gram`s staining, coagulase production, resistance to oxacillin, and polymerase chain reaction (PCR) amplification of *coA* [28], *nuc* [48]*,* and *mecA*[2] genes. PCR for the aforementioned genes was performed using Applied Biosystems SimpliAmp Thermal Cycler (ThermoFisher Scientific, USA) and PCR conditions suggested by [28, 48] and [2]. All the isolates were stored at –80 ℃ in trypticase soy broth with 20% (v/v) glycerol for further analysis. The antimicrobial susceptibilities of all MRSA isolates were detected using the Kirby-Bauer disk diffusion method on Mueller–Hinton agar plates (HiMedia laboratories Pvt Ltd., Mumbai) according to the guidelines of the Clinical and Laboratory Standards Institute (CLSI) [11]. The following antibiotic disks (HiMedia laboratories Pvt Ltd., Mumbai) were used: oxacillin (1 μg), cefoxitin (30 μg), erythromycin (15 μg), azithromycin (15 μg), tetracycline (30 μg), trimethoprim-sulfamethoxazole (25 μg), levofloxacin (5 μg), ciprofloxacin (5 μg), gentamicin (10 μg), clindamycin (2 μg), chloramphenicol (30 μg), linezolid (30 μg), and nitrofurantoin (300 μg). Tested strains were evaluated as susceptible or resistant by measurement of their zones of inhibition and were defined as multidrug-resistant (MDR) if they exhibited resistance to at least one agent in three or more antimicrobial classes [36]. *S. aureus* ATCC 25923 was used as quality control strain.

### Whole genome sequencing, assembly, and annotation

The QIAamp DNA Mini Kit (Qiagen, UK) was used to extract the genomic DNA of the 36 MRSA isolates according to the manufacturer instructions with some modifications to facilitate obtaining pure DNA of concentrations greater than 50 ng/μg. These modifications include increasing the initial volume of bacterial suspension to 2 ml, and warming the elution buffer to 60 ℃. Moreover, 200 μl of 20 mg/ml lysostaphin (Sigma-Aldrich, Switzerland) containing lysis buffer was added to facilitate cell lysis. DNA was eluted in 100 μl of elution buffer and quantified using a Nanodrop spectrophotometer. Libraries were prepared using NexteraXT kits (Illumina, San Diego, CA) according to manufacturer’s instructions and batches of 48 isolate gDNA were barcoded. MiSeq run kits (v3) were used to generate 2 × 300 base paired-ends on Illumina’s MiSeq sequencing platform. Fastp command line tool was used for quality control, adapter trimming, and quality filtering of the FASTQ data with the activated error correction option using C parameter to perform overlap analysis for paired end reads [10]. SPAdes (version 3.13.0, using the –careful command) was used to de novo assemble the genome [10]. Then, assembly statistics are calculated on the filtered assembly with QUAST 4.4 [26]. Assemblies were polished 5 times using Pilon [55]. Average polished assemblies were 2534291.639 bp (Additional file 1). The assembled contigs were processed using Prokka v1.14.5. [50] for annotation of sequences and prediction of genes using a core set of conserved prokaryotic genes. then, PEPPAN, a pan genomics tool that provides consistent annotation and paralogs exclusion using combining tree- and synteny-based approaches, was utilized for pangenome analysis [59]. Genes were considered as core if they present in 95% of the genomes (Additional file 2).

### Multilocus sequence typing, SCC*mec* and SPA typing

FastMLST, a multilocus sequence typing tool that uses BLASTn to perform PubMLST searches [25], was used to determine the ST, allelic profile and clonal complex for the draft assemblies [25]. Staphopia-sccm command line tool was executed to determine the SCC*mec* types of the 36 HA-MRSA samples from their draft assemblies [44]. In addition, SpaTyper (https://github.com/HCGB-IGTP/spaTyper, Version 0.3.3) was utilized to generate spa type identification from the draft assemblies.

### In silico identification of resistome and virulome

ABRicate (https://github.com/tseemann/ABRicate Version0.7) was used to screen the assemblies for AMR genes with NCBI Bacterial Antimicrobial Resistance Reference Gene Database [10]. In addition, ABRicate v0.7 was also utilized to screen for virulence factor using Virulence Factor Database (VfDb) [10].

### Phylogenic and recombination analysis

Parsnp from Harvest v1.2 was utilized to construct core genome alignments [54]. The contiguous non-conserved regions of the alignment file was removed using Gblocks v0.91b [9]. RAxML-HPC v8 command line tool with a general time reversible model, GAMMA substitution rate and rapid bootstrapping (*n* = 1000) was used to construct the maximum-likelihood (ML) phylogeny. The iTol [33] tool was used to visualize the resultant RaxML best tree and it’s meta data. ClonalFrameML v1.11 was utilized to determine the recombination in the core genome with -emsim set at 100 simulations [16] using the RaxML produced tree as a starting tree and Parsnp core genome alignment.

### Correlation matrix analysis:

The data of antibiotic resistance (AR) genes, antimicrobial resistance profile (phenotype), and clonal complexes (CC) were first transformed into binary data (0 and 1) where 0 refers to antibiotic susceptibility, AMR gene absence, or CC absence while 1 represents antibiotic sensitivity, AMR gene or CC presence. The data was uploaded on R software (version 4.2.1, https://www.r-project.org, accessed on 1 May 2023). Pearson’s correlation coefficients were then calculated using the cor function. Then, the significance of the correlation data was estimated using the cor.test function. Finally, only the significant correlations (P < 0.05) were demonstrated in a visualized correlation matrix by the corrplot function.

## Results

### Genome features

The average genome sizes of MRSA isolates were 2.53 ± 0.1 Mbp with GC content 33.4% (Additional file 1). N50 ranged from 12589 to 1844 bp and the number of contigs ranged from 1448 to 486 contig. Pangenome analysis showed that 1204 genes were considered as core genes, 589 cloud genes, and 443 shell genes. Core genes occupied 53.8% of genome length, whereas 26.3% and 19.8% were cloud and shell genes, respectively (Additional file 2).

### Population structure of Egyptian HA-MRSA isolates

**Fig. 1:**
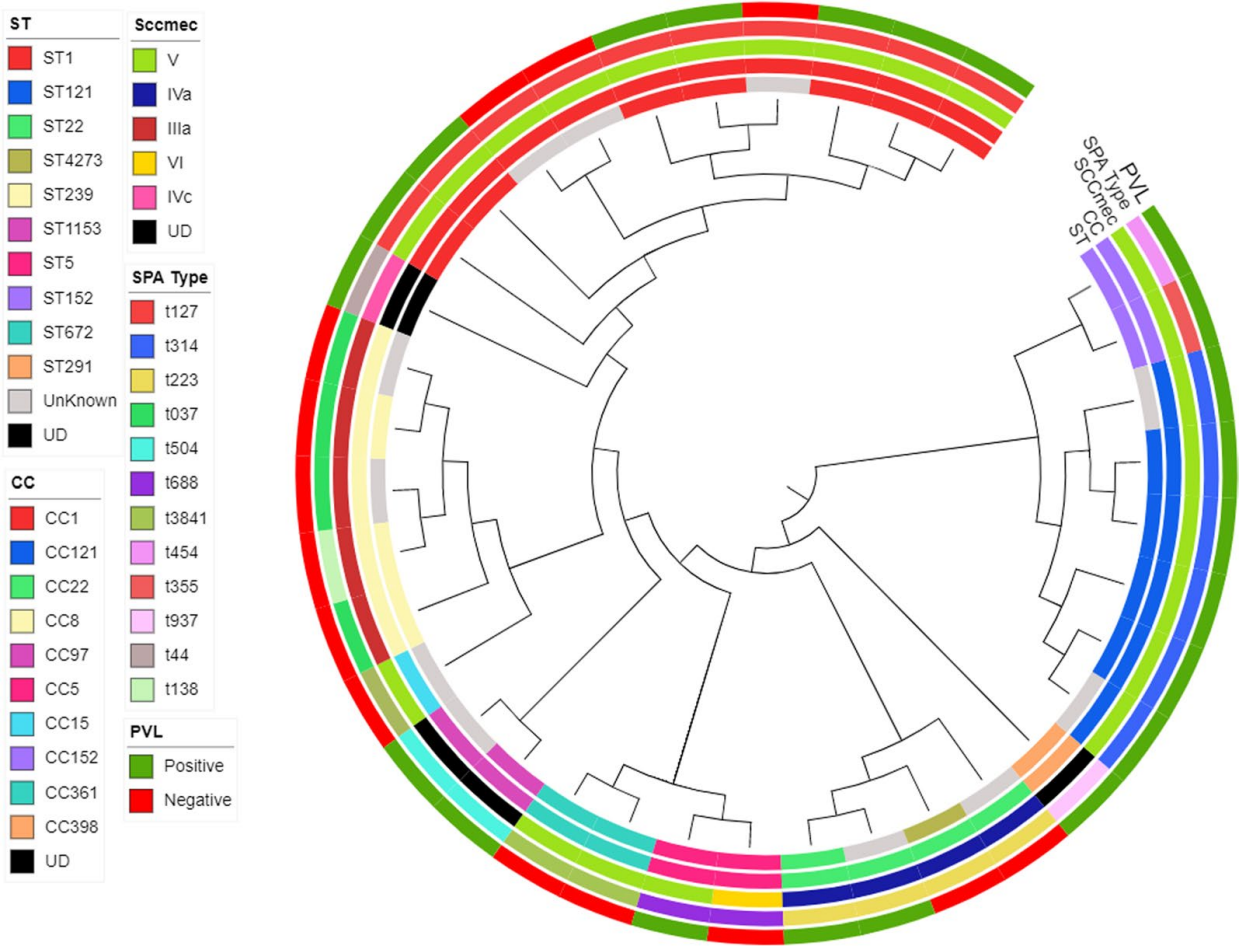
Maximum-likelihood (ML) phylogeny of 36 HA-MRSA isolates recovered from Egyptian health care settings. The phylogenetic tree was constructed based on core genome SNPs using Parsnp tool and Gblocks v0.91b to remove contiguous non-conserved regions from the alignment. CIPRES-hosted RAxML-HPC v8 was utilized to construct the ML phylogeny from the core genome alignment with a General Time Reversible model, GAMMA substitution rate and rapid bootstrapping (*n* = 1000). *iTOL* was utilized for visualization of the ML tree and the Meta data. Meta data includes ST types, clonal complexes; Spa types, SCC*mec* type, and PVL presence are color-coded in the inner rings

In silico multi-locus sequence typing (MLST) was performed to characterize clonal distribution of the screened isolates. A total of 10 sequence types (STs) belonging to 10 clonal complexes (CCs) were identified (Fig. 1). The most common sequence types in our MRSA collection are ST1 (N = 7/36; 19.4%), ST121 (N = 4/36; 11.1%), and ST239 (N = 3/36; 8.3%). Eleven isolates had an undetermined sequence type. The four most prevailing CCs (CC1, CC121, CC8 and CC22) comprised more than 69% of screened isolates. The isolates belonged to 14 *spa* types. The dominant *spa* type was t127 that was detected in 10 (27.8%) isolates. Other major *spa* types were t314 (N = 6; 16.7%), t037 (N = 4; 11.11%), and t223 (N = 3; 8.3%). Some *spa* types were observed to present sporadically including t504, t688, t3841, t138, t84, t454, t355, t937 and t44. One isolate had unknown *spa* type with unidentified repeat succession (26-23-13-23-05-25-17-25-23-28).

Despite the observed diversity of ST types and *spa* types, the majority of isolates (N = 22; 61%) harbored SCC*mec* type V, a type previously reported to be associated with community acquired clones. SCC*mec* type V was harbored by isolates with diverse clonal complexes including CC1, CC121, CC5, CC15, CC152, and CC361. Other prevalent SCC*mec* types included IIIa (N = 5; 13.8%) and IVa (N = 4; 11.1%). On the other hand, SCC*mec* types IVc and VI were detected once. Unknown SCC*mec* pattern was observed in three isolates.

To further explore specific lineages with distinct genotypes, the results of the previous characterization methods were combined. Almost one third of isolates (N = 10; 27.8%) belonged to the ubiquitous CA-MRSA lineage, CC1-SCC*mec* V. All isolates in this lineage belonged to t127 *spa* type. The globally disseminated hypervirulent clone CC121-SCC*mec* V PVL + was the second most prevalent genotype (N = 6; 16.7%). Although *spa* types associated with CC121 clone are quite diverse [46], t314 was the only *spa* type detected in this clone in our study. Interestingly, the widely spread hospital associated CC8-ST239-MRSA-III clone was only found in 8.3% of screened isolates (Fig. 1).

### Association between AMR markers and clones with resistance phenotypes

We evaluated antibiotic resistance phenotypes among screened HA-MRSA isolates. All MRSA isolates were susceptible to nitrofurantoin. More than half of isolates (58.3%) were classified as multidrug resistant (MDR) as they were non-susceptible to at least three different classes of antibiotics (Table 1). An association between clonal lineages and MDR was observed. For instance, the predominant CC1 showed a high rate of multidrug resistance (80%) compared to other major clonal complexes. On the other hand, the second most abundant clonal complex, CC121, showed a lower MDR rate of 33.3%. Alarmingly, four isolates (11.11%) of different clones (CC1, CC8, and CC361) were resistant to linezolid, a last resort antibiotic in treating multidrug resistant MRSA [47]. Apart from the aforementioned antibiotics, chloramphenicol and tetracycline displayed the best antimicrobial activity (83.3% and 75% sensitivity among screened isolates), while gentamicin exhibited the weakest activity (13.8% sensitivity). Furthermore, we observed 52.7% and 41.6% sensitivity rates towards fluoroquinolone and macrolide antibiotics, respectively. It’s noteworthy that among our three major clonal complexes, CC121 isolates were susceptible to most antibiotics used, whereas CC1 and CC8 showed higher rates of phenotypic resistance. The latter two clonal complexes showed comparable rates of fluoroquinolone and macrolide resistance (Table 1). Interestingly, although trimethoprim-sulfamethoxazole was active against MRSA in this study (66.6% sensitivity), half of trimethoprim-sulfamethoxazole resistant isolates belong to CC1. Interestingly, different clones were displaying heterogeneous AMR patterns.

**Table 1:** Antimicrobial resistance patterns and clonal complexes among HA-MRSA isolates

AMR profile^a^	Number of Isolates (%)	Clonal Complex
**GEN ERY CD CIP COT C LZ**	4 (11.11%)	CC1 (2), CC8 (1), CC361 (1)
**GEN ERY CD CIP COT**	4 (11.11%)	CC1(2), CC97 (1), UD CC (1)
**GEN ERY CIP COT**	3 (8.33%)	CC1 (1), CC22 (1), CC97 (1)
**GEN ERY CD CIP TET**	1 (2.77%)	CC121 (1)
GEN	12 (33.33%)	CC1 (2), CC121 (3), CC8 (2), CC5 (1), CC152 (2), CC361 (1), CC398 (1)
**GEN TET**	4 (11.11%)	CC1 (1), CC121 (1), CC5 (1), CC15 (1)
**GEN ERY**	2 (5.55%)	CC1 (1), CC22 (1)
**COT C**	1 (2.77%)	CC1 (1)
**GEN ERY CIP**	1 (2.77%)	CC8 (1)
ERY	1 (2.77%)	CC22 (1)
**ERY C TET**	1 (2.77%)	CC8 (1)
None (pan susceptible)	2 (5.55%)	CC121 (1), CC22(1)

^a^MDR strains are in bold and underlined

**Fig. 2:**
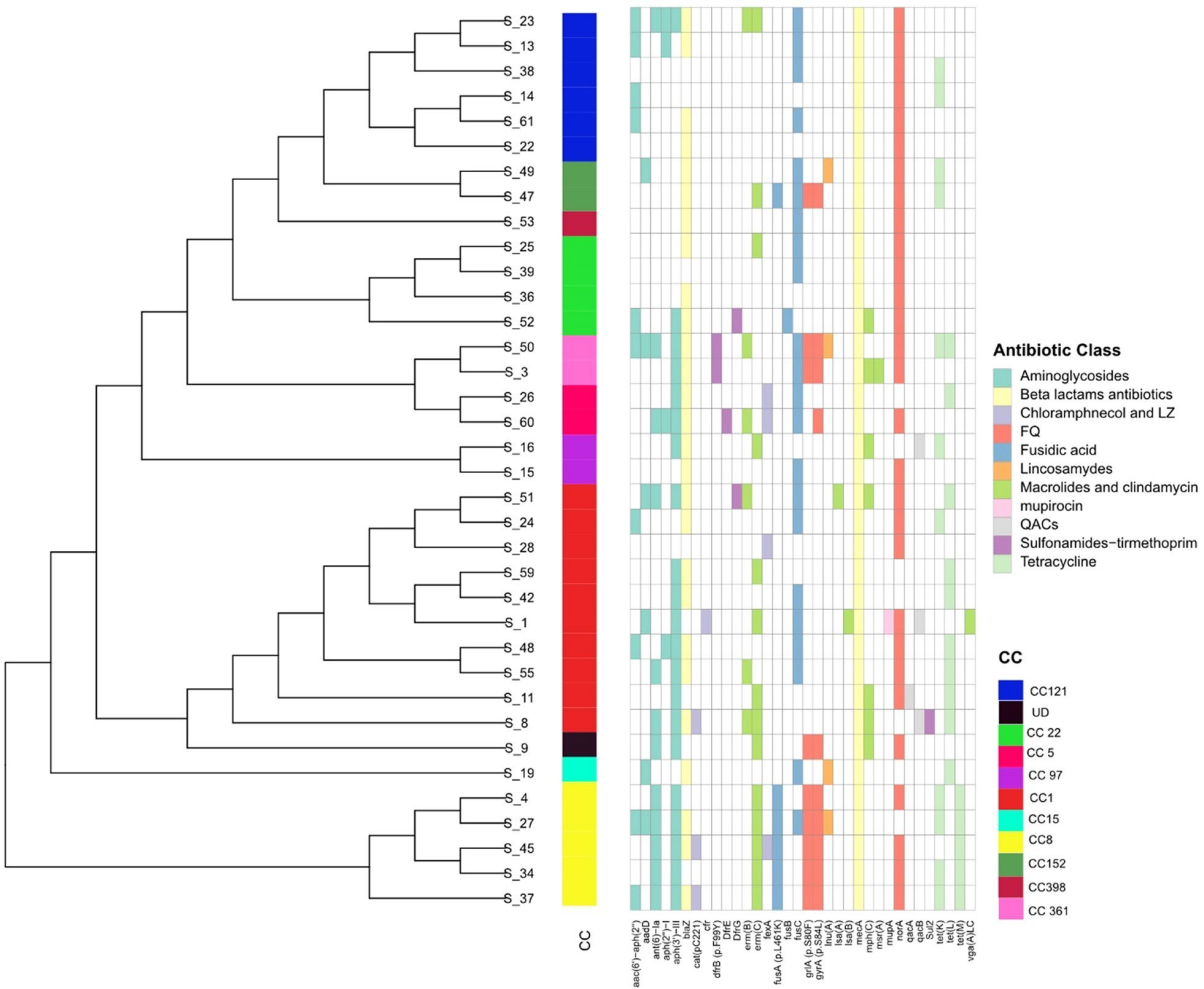
Antibiotic resistance profiles and association with resistance determinants and clonal complexes of HA-MRSA. ITOL web tool was utilized to visualize the Maximum-likelihood phylogenetic trees of 36 HA MRSA with their distribution of antibiotic resistance determinants (acquired genes and point mutations) relative to different clonal complexes, the clonal complexes for all samples were coded using a color strip in iTOL

Next, we determined the association between different resistance determinants, including acquired genes and gene mutations, with phenotypic resistance (Fig. 2). As expected, in-silico resistome analysis showed that all isolates harbored *mecA* gene conferring resistance to methicillin and other beta-lactam antibiotics. The *mecC,* a divergent form of *mecA*, was not detected. Another penicillin resistance encoding gene, *blaZ*, was present in 75% of the screened isolates. Regarding non-β lactam antibiotics, we found 30% discordance between phenotypic and genotypic resistance (Additional file 3). This discrepancy might be attributed to unexpected or unknown resistance genes or alternative resistance mechanisms that warrant further study using genome wide association studies on larger number of screened isolates. The multidrug efflux pump gene, *norA*, known to contribute to fluoroquinolones resistance, was widespread in our dataset as it was detected in 80.6% of screened isolates. Besides *norA*, mutations in the quinolone-resistance determining regions (QRDR) of the *grlA* and *gyrA* genes were found to lesser extent. Notably*, grlA* and *gyrA* genes mutations were present in all CC8 isolates, but completely absent in other major CCs (CC1, CC121 and CC22). This suggests that MRSA clones’ fitness might be influenced by the presence of such nucleotide mutations [30]. Regarding macrolides antibiotics, ribosomal target modification mediated by *erm* genes was observed to be the primary resistance mechanism. The *ermC* and *ermB* genes were found in 38.8% and 16.67% is screened isolates, respectively. On the other hand, *ermA* was absent in our dataset. Additional macrolide resistance gene, *mphC*, was sporadically present in the screened isolates.

Despite phenotypic susceptibility to tetracycline, more than half of isolates harbored at least one tetracycline resistance gene. Three tetracycline resistance genes were present: *tetM, tetK,* and *tetL*. Chromosomal or transposal *tetM* mediates resistance via encoding a ribosomal protection mechanism. On the other hand, *tetL* and *tetK* are located on plasmids and encode tetracycline efflux pumps. Interestingly, *tetM* was exclusively present in CC8 isolates, while the majority of *tetL* genes (70%) were found in CC1. Aminoglycoside resistance genes were distributed among the isolates, with *aph(3')-III* being the most prevalent and present in 58.8% of the samples. Although we observed high phenotypic resistance towards gentamicin, *aac(6')-aph(2'')* and *aph(2'')-Ia* genes, associated with gentamicin resistance, were detected at low frequencies (27.7% and 11.11%, respectively). Finally, we observed the presence of fusidic acid resistance determinants, particularly *fusc*, that was detected in 61.1% of screened isolates. Nonetheless, *fusB* was only detected once. Furthermore*, fusA* (p.L461K) gene mutation was detected in 16.6%, and was mostly associated with CC8 clone.

### Virulome analysis

**Fig. 3:**
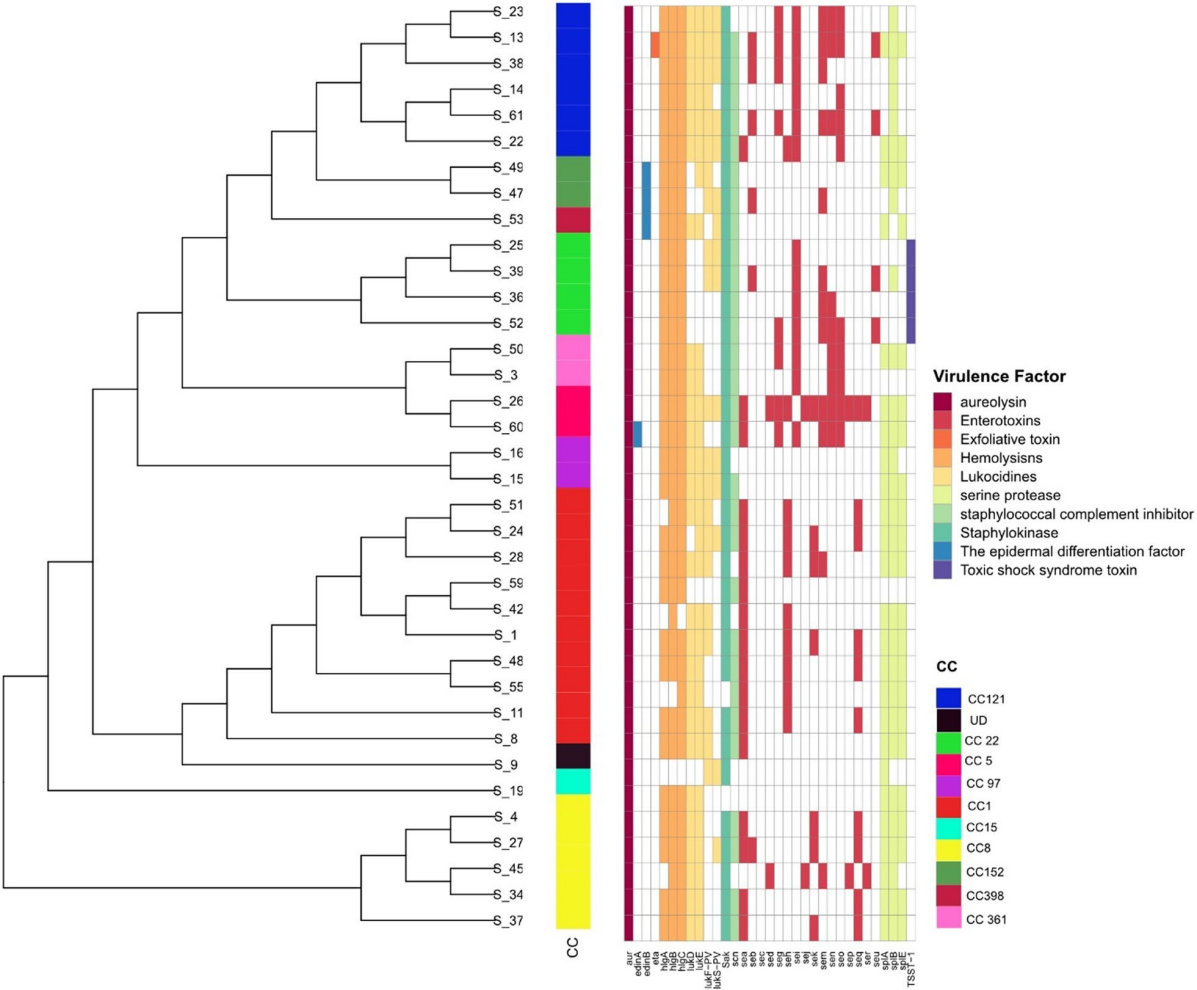
Significant virulence encoding genes profiles of the 36 HA-MRSA isolates. ITOL web tool was utilized to visualize the Maximum-likelihood phylogenetic trees of 36 HA MRSA with their distribution of virulence factors and superantigens in HA-MRSA isolates relative to different clonal complexes. Clonal complexes for all samples were annotated using a color strip in iTOL

Virulome analysis revealed the ubiquitous distribution of virulence-related genes among our MRSA collection (Fig. 3). The total number of virulence factors detected using VFDB ranged from 26 to 58 genes per isolate. All isolates were positive for *aur* gene encoding aureolysin, and at least one gamma hemolysin encoding gene. Another important virulence factor is the Panton-Valentine leukocidin (PVL) pore-forming cytotoxin. This is a bicomponent toxin encoded by *lukF*-PV and *lukS*-PV genes and is often linked to community acquired MRSA strains. Among our collection, we detected the presence of *lukF*-PV and *lukS*-PV genes in 21 (58.8%) and 14 (38.8%) genomes, respectively. Notably, PVL genes were found in almost all clonal complexes except for CC8, CC15, and CC361 isolates, which were PVL negative. Other prevalent leucocidin encoding genes include *lukE* and *lukD* which were detected at 80.5% and 75% frequencies, respectively. Serine proteases were also widespread among our isolates, with SplB encoding gene being present in 29 genomes (80.5%), whereas SpIA and SpIE encoding genes are detected in 25 (69.4%) and 22 (61.1%) of isolates, respectively. Noteworthy, most of isolates, regardless of their clonal complexes, carried a wide array of staphylococcal enterotoxins encoding genes (Fig. 3). Toxic shock syndrome toxin encoding gene (TSST-1) was exclusively present in all CC22 isolates, while exfoliative toxin gene *eta* was only detected once in CC121. Other exfoliative toxin genes were not detected in our dataset.

### Distribution of AMR determinants and virulence genes among MRSA clones

**Fig. 4:**
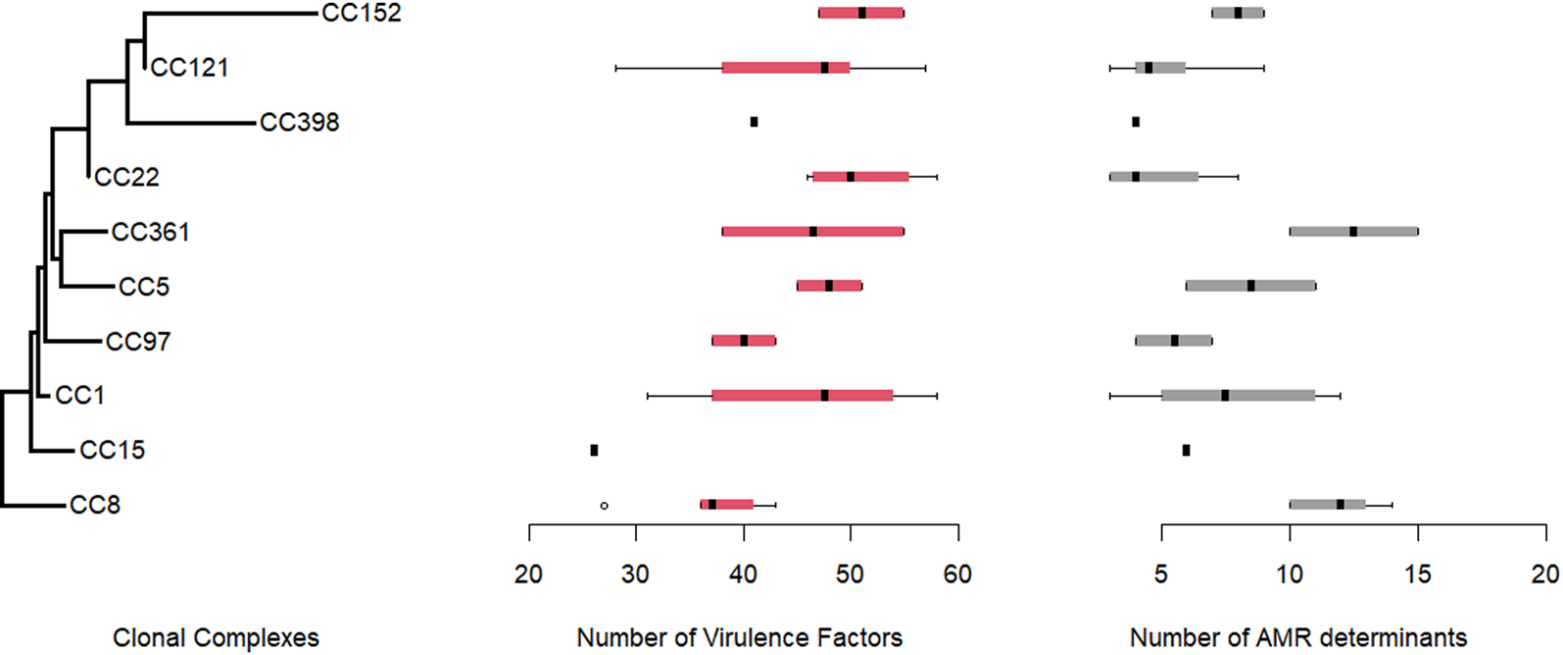
Summary boxplots of the number of virulence factors identified using the VfDb and antimicrobial resistance identified using NCBI identified in each clonal complex. All data points shown, bars show min and max. This plot was made utilizing graphics R package (Version 4.1.1) and ape package (Version 5.7.1)

Quantification of resistance and virulence determinants revealed that the genome of CC1 (a community-lined clone) harbored a high number of both AMR determinants and virulence factors encoding genes, suggesting the co-occurrence of extensive AMR in a hypervirulent genomic background among this hospital-adapted clone (Fig. 4). In silico analysis revealed that the overall number of AMR determinants in the genomes of CC1 isolates ranged from 3 to 11, while virulence encoding genes ranged from 31 to 54. To highlight the factors underlying the expansion of CC1 isolates in the hospital settings, we mapped the presence and absence of resistance and virulence genes among the four major clones detected in this study (Additional file 4). Multiple resistance genes associated with resistance to aminoglycosides, fluoroquinolones, and macrolides were shared among the four clones. Genes conferring resistance to quaternary ammonium compounds (*qacA* and *qacB*) were exclusively present in CC1 (Additional file 4), which might have granted this clone a competitive advantage in the hospital environment. Mutations in *gyrA*, *grlA*, and *fusA* genes were only present in CC8, whereas the *fusB* gene was only detected in CC22. Virulence genes, on the other hand, were broadly shared among the four clonal complexes. Most CCs carried more than one enterotoxin, hemolysin, and seine protease encoding genes. Nevertheless, Some virulence genes were found in certain clonal complexes, including the toxic shock syndrome toxin encoding gene in CC22, the exfoliative toxin encoding gene in CC121 and genes encoding for staphylococcal enterotoxins r and p in CC8.

### Increased recombination in CC1 and CC8 core genomes

**Fig. 5:**
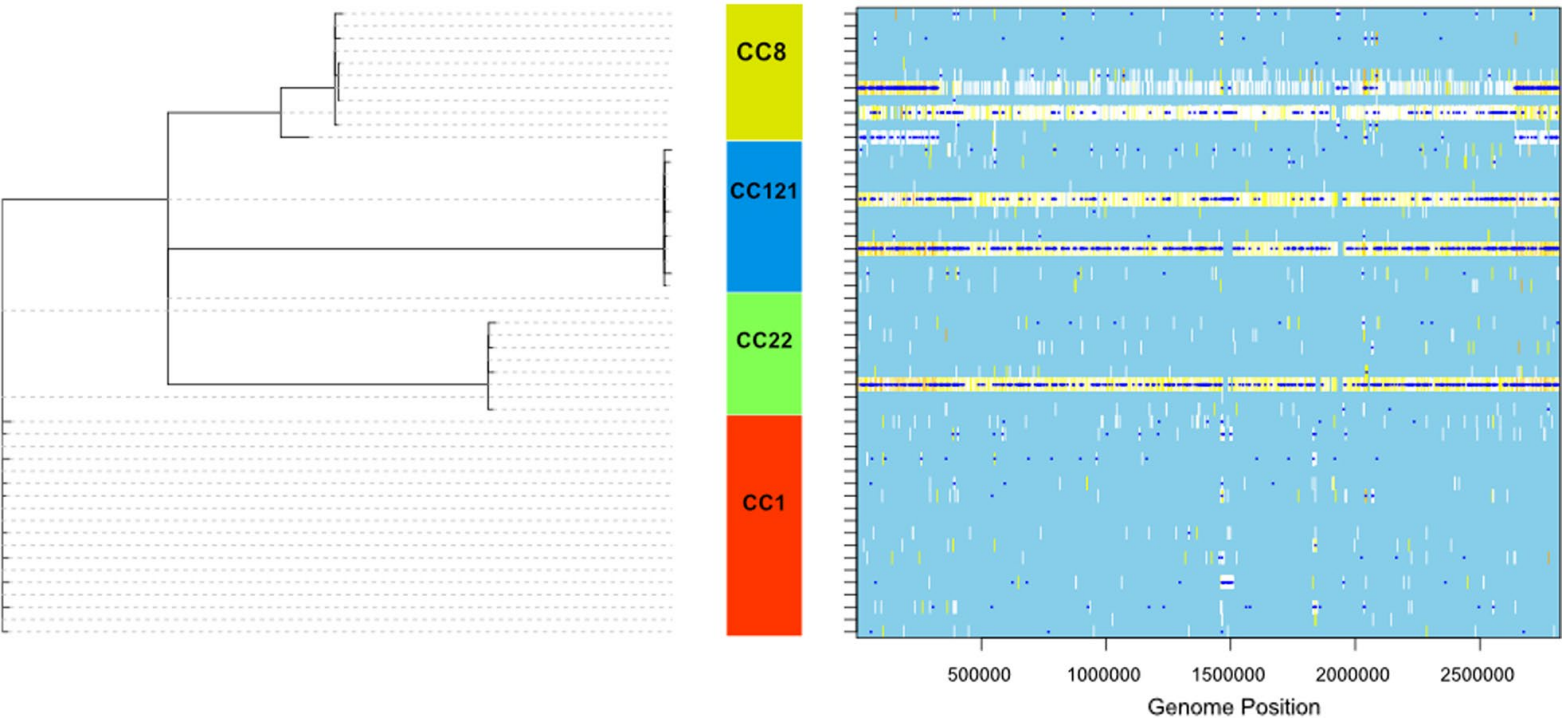
Clonal Frame analysis of the recombination in the core genome of the major clonal complexes in 36 HA-MRSA isolates. Dark blue horizontal bars indicate recombination events detected by the analysis; Light blue represent sites that are non-polymorphic for a given branch while white color shows that there is no homoplasy

Parsnp tool [54] was utilized to obtained core genome alignment between all HA-MRSA isolates that were assigned to CC1, CC8, CC121, and CC22. Then, RAxML [53] was utilized to construct a maximal likelihood phylogeny from the core genome alignment of all samples. ClonalFrameML used the Parsnp alignment and RAxML tree to analyze the recombination in the major clonal complex samples revealed in this study. For all clonal complexes together, the rate at which recombination introduced nucleotide changes, relative to mutation (r/m) was 1.79 (95% CI 1.77–1.80, suggesting that the effect of recombination among these isolates was almost double the de novo mutations. Furthermore, using further analysis by clonal frame, we compared the recombination events among the most prevalent clones in this study (CC1, CC8, CC121, and CC22). Interestingly, CC1 had the highest R/theta (0.239) which indicates the ratio of frequency of recombination and mutation, with average the recombination events length of 118.94 bp (1/delta = 0.008), producing r/m = 1.43, which indicates that the relative effect of recombination was more than the effect of mutation. Additionally, CC8 had the highest r/m = 8.56 which due to the increased average length of recombination events = 2863.26 bp while it’s R/theta = 0.142. Finally, CC22 also had a higher effect of recombination than mutation with r/m = 1.32 with average insert length of 105.51 bp. On the other hand, CC121 had a relative effect of recombination less than mutation with r/m = 0.92 (Fig. 5).

### Correlation between genotypic and phenotypic AMR

**Fig. 6:**
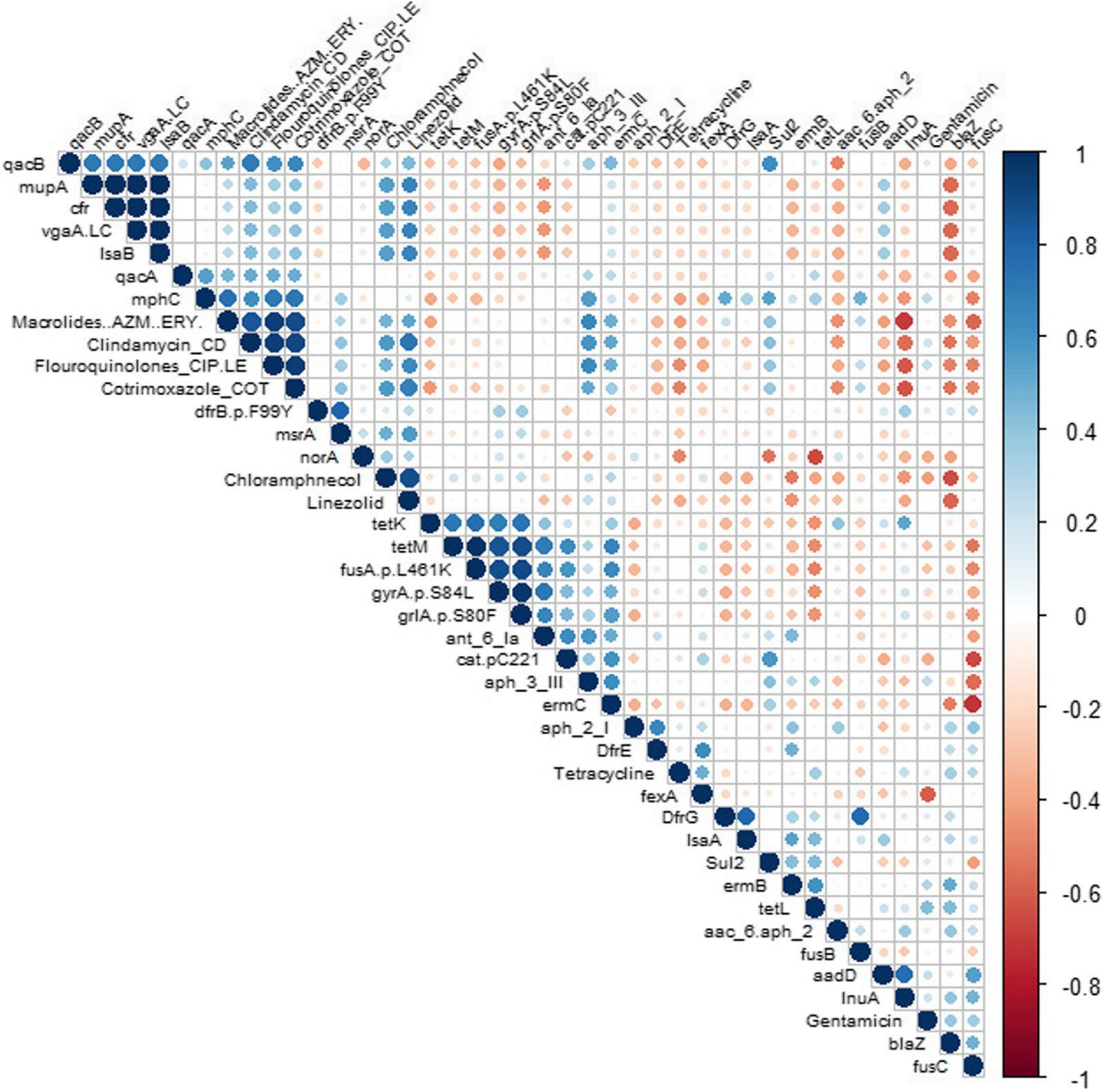
Correlation matrix between antibiotic resistance genotype and phenotypic features with the statistically significant correlations (p <  = 0.05). The white squares show no significant correlation. Red dots show a significant negative association, while blue dots show a significant positive correlation. The sizes and the color of the dots degrees are matched to the correlation coefficient's value (r)

A Correlation analysis was conducted to determine the co-resistance pattern between different phenotypic and genotypic traits (Fig. 6) and to detect the existent associations between different resistance determinants and clonal complexes (Additional file 5). The correlation analysis indicated a positive correlation between phenotypic resistance towards macrolides, clindamycin, fluoroquinolones and cotrimoxazole. Tetracycline resistance, on the other hand, was negatively correlated with the aforementioned antibiotic classes. Notably, despite the small number of linezolid-resistant isolates, phenotypic resistance to linezolid was positively correlated to resistance to most antibiotics used (except tetracycline), indicating the potential of these isolates to develop pan-resistance. Among the observed negative correlations is the one between the ubiquitous *norA* gene and *tetL* gene, which may explain the negative correlation between fluoroquinolones and tetracycline resistance. Other negative correlations include the ones between *fusC* and *ermC* and between *blaZ* and chloramphenicol resistance. Regarding the correlations between clonal complexes and antibiotic resistance (Additional file 5), the predominant CC1 was negatively correlated to *gyrA*, *grlA,* and *fusA* gene mutations and positively correlated to *qacB* and *tetL*. On the other hand, CC8 was negatively correlated to *tetL* but positively correlated to the previously mentioned gene mutations.

## Discussion

Ever since its first description, MRSA has been known for its exceptional adaptability and evolving mechanisms into endemic and epidemic strains conferring a serious threat to public health. MRSA epidemiology varies in different geographical locations and healthcare settings [4]. Typically, in each location, few MRSA clones predominate for a while before declining and being replaced by new successful strains [32]. Therefore, monitoring the local and global molecular and genomic epidemiology of MRSA is crucial to understand the drivers of resistance spread and to direct antibiotic stewardship policies in local hospitals.

In this study, despite the limited number of isolates, MRSA STs and CCs were quite diverse, indicating weak clonality and high dissemination of Egyptian isolates. The most frequently detected sequence types were ST1 (19.4%), ST121 (11.1%), and ST239 (8.3%). Twelve isolates were missing one or two alleles of the seven housekeeping genes and had an undetermined sequence type. Clonal complexes were determined based on sharing five or more alleles with the central genotype (ST). The results of CC typing were confirmed by maximum likelihood phylogenetic analysis, which demonstrated that isolates belonging to the same clonal complexes were closely related. According to Durand et al., Wg-MLST is particularly liable to genome fragmentation which disables marker calling [18]. Phylogenetic analysis is less sensitive to this issue; therefore, it can be used to conclude the closely related genotypes. Regarding CC types, the community associated CC1 clone was predominating in our hospital-recovered isolates comprising about third of the isolates (27.8%), followed by CC121 (16.6%) and CC8 (13.8%).

Previous studies on MRSA from Egyptian health care settings suggested temporal and spatial epidemiological variation. For instance, ST1535-V, ST1-V and ST239-III comprised the majority of MRSA isolates obtained from patients admitted to major tertiary hospital in Cairo between 2017 and 2018 [52]. Another study revealed that CC15-V and CC1-V were the predominating clonal complexes in a hospital in Alexandria in 2020 [37]. Previous surveillance studies indicated that ST239-III (CC8), the Brazilian/Hungarian clone, was the most commonly detected HA-MRSA clone in multiple African and Middle Eastern cities [32]. Also, a recent Egyptian study showed that different variants of the ST239-MRSA-III clone were comprising the majority of their MRSA isolated from hospital in Alexandria between September and December 2015 [38]. Nonetheless, although our isolates are epidemiologically classified as HA-MRSA, only 8.3% belong to ST239-MRSA-IIIa strain.

One of the most important findings of this study is the remarkable expansion of the community-linked clone CC1 in the Egyptian healthcare settings. Lately, CC1 has been identified as a very successful CA-MRSA lineage [32]. MRSA strains belonging to this clone usually carry SCC*mec* type IV or type V (5C2) that are also more linked to CA-MRSA [32]. CC1 has been recently reported to be associated with multiple outbreaks in hospitals in different geographical locations. For instance, multiple strains of CC1 with MDR profiles were spreading among different Irish hospitals [19]. Similarly, CC1 was reported in nosocomial outbreaks in many European countries [45, 52]. Furthermore, CC1 have emerged in livestock animals through human-to-animal host jumps, giving rise to livestock-adapted clones [3, 21]. In this study, nearly one-third of the isolates belong to CC1. All isolates belonging to CC1 are associated with SCC*mec* type V and *spa* type t127, and 70% were PVL-positive indicating the dissemination of CA-MRSA in some Egyptian hospitals. These results suggest the adaptation of CC1 clone to survive in extreme antimicrobial selective pressure encountered at the Egyptian health care settings that was triggered by the improper use of antibiotics in hospitals. Previous epidemiological studies reported that multiple CA-MRSA lineages displacing conventional HA-MRSA strains in healthcare settings worldwide. For instance, the nosocomial outbreaks caused by the pandemic strain USA300 (ST8-IV lineage) are one of the earliest examples of CA-MRSA invading hospitals in North America [33, 34]. USA300 replaced USA100 (ST5-II lineage) in multiple hospitals in New York City. In China, ST59, again, a major CA-MRSA lineage circulating in East Asia, is gradually taking over ST239 and ST5 clones in Chinese hospitals [30, 34, 42]. Mathematical models suggest that CA-MRSA will competitively exclude HA-MRSA in healthcare facilities and eventually displace it [13]. This phenomenon might lead to devastating consequences, especially in low resources Countries like Egypt. Two possible mechanisms explain the adaptation success of CA-MRSA in hospitals. One mechanism is mediated by the intrinsic physiological properties of the successful strain; for instance, better capacity to survive outside the host [34]. The other mechanism is through gaining resistance to antimicrobials. In this study, adaptation of CC1 clone to the hospital settings was reflected on the AMR phenotypic trains as shown by high multiple drug resistance rates (80%). Furthermore, analyzing the resistome and virulome of CC1 isolates revealed a high pool of AMR resistance determinants and a higher content of virulence-related genes. Such expansion of CC1 from the community to the hospital setting might be triggered by the pressure imposed by misuse of antibiotics that consequently selects for highly resistant and virulent isolates.

Recombination analysis was conducted to underline mechanisms driving the evolution and dissemination of specific MRSA lineages in the hospital environment. Generally, the recombination rates of different MRSA clones or even different lineages in the same clone vary depending on multiple factors. In this study, we observed an overall high recombination rate relative to mutation (1.79). CC1 had the greatest recombination frequency among the four major clones. On the other hand, the greatest length of recombination events was observed among CC8 isolates. Castillo-Ramírez et al*.* found that recombination rates of MRSA strains vary dramatically with geographical location giving each location its distinct population structure [8]. They found that the relative recombination to mutation rates of the hospital-acquired MRSA clone ST239 varied greatly between South America, Asia and Turkey. According. Driebe et al*.,* despite the little recombinogenic properties of *S. aureus*, recombination occurs in epidemic lineages that expand in a clonal manner [17]. The high recombination frequency of CC1 might be facilitated by its ability to produce biofilms and provide a suitable environment for horizontal gene transfer events [8]. The high rate of recombination observed in CC1 might have played an important role in horizontal acquisition of resistance genes and subsequently its broad dissemination, and can lead to continuous endemics in hospital settings and even enhanced growth in different unexpected ecological niches [51].

Regarding SCC*mec* types, the majority of the isolates harboured SCC*mec*-V, which is usually associated with CA-MRSA infections. Notably, these isolates were highly diverse, suggesting that SCC*mec*-V spread is not associated with a single outbreak clone. MRSA-V and MRSA-IV carry smaller SCC*mec* elements compared to MRSA-II and MRSA-III [56]. According to a previous study, smaller SCC*mec* cassettes might be associated with lower fitness costs [29]; thus, the spread of clones carrying SCC*mec*-V may be attributed to its reduced fitness burden.

Another concerning observation in this study is that phenotypic linezolid resistance observed among 11.11% of our tested isolates. Unfortunately, linezolid has been extensively prescribed during the COVID-19 pandemic in Egypt for presumptive patients [20]. Resistant isolates belong to different clones (CC1, CC8 and CC361), suggesting that linezolid resistance is not necessarily associated with MRSA success in hospitals. Alarmingly, all linezolid resistant isolates were phenotypically resistant to all antibiotics used in this study except tetracycline and nitrofurantoin. Linezolid resistance was recently reported in the United Arab Emirates and Kuwait [49]. Recent Egyptian study reported the emergence of linezolid resistance in MRSA which was accompanied by high biofilm producing capability [1]. The emergence of linezolid-resistant MRSA strains in the Middle East is distressing as it will limit treatment options available for multidrug-resistant MRSA.

Regarding resistome analysis, *norA* is the most prevalent non-beta-lactam resistance gene in our dataset. This gene encodes a multidrug efflux pump and is associated with resistance against fluoroquinolones, several disinfectants, and antiseptics [12] and is suggested to play a role in MRSA survival in harsh disinfection conditions in the hospital. On the other hand, *gyrA* and *grlA* gene mutations were not frequently detected and were mainly associated with CC8 and CC361 isolates. Regarding erythromycin resistance, *erm(C)* was the most detected resistance determinant, with the sporadic presence of *erm(B)* and complete absence of *erm(A)* genes. We also observed a high prevalence of tetracycline resistance genes; *tetM* was exclusively present in CC8 isolates, while *tetL* was mainly associated with CC1. Finally, the *fusC* gene encoding fusidic acid resistance was widely spread among screened isolates. This is not surprising as fusidic acid is readily available as an over-the-counter medication in Egyptian pharmacies and is usually used in multiple skin conditions without any prescription [37].

One of the limitations of this study is the limited number of isolates. Moreover, MRSA typing and characterization of resistance and virulence determinants was mainly dependent on existing databases which might explain the genotypic-phenotypic discrepancies encountered.

In conclusion, CC1-MRSA, an emerging CA-MRSA strain, has gained a foothold in healthcare settings. Although CA-MRSA clones are usually less resistant than HA-MRSA, the selective pressure posed by antimicrobial use in hospitals may end up with extensive AMR in a hypervirulent genomic background. The capability of CA-MRSA to cause nosocomial infections compels us to reconsider their increasingly blurring definition. Since prevention is better than cure, serious measures should be taken to limit the dissemination of AMR among MRSA to avoid devastating consequences. WGS is powerful for regular surveillance of MRSA characteristics and evolution and hence directing efforts towards controlling it. Unless appropriate strategies are taken to direct antibiotic use in hospitals, MRSA may pose a considerable threat to humans in the future. Follow up surveillance studies should continue monitoring the geographical long-term dynamics of MRSA in Egyptian hospitals. Future studies should also focus on elucidating the AMR patterns and clinical outcomes associated with high-risk clones to enable updating existing antibiotic stewardship programs as well as predicting disease outcomes.

### Supplementary Information

**Additional file 1. **Assembly statistics for All 36 HA-MRSA samples generated using Quast tool.

**Additional file 2. **Pie chart for Pangenome analysis, 1204 gene were considered as core genes, 589 cloud genes, and 443 shell genes.

**Additional file 3. **Typing results and AMR genotypic/phenotypic concordance and discordance.

**Additional file 4. **Distribution of antibiotic resistance profiles (**A**) and virulence (**B**) genes in the four major clonal complexes (CC1, CC121, CC8 and CC22).

**Additional file 5. **Correlation matrix between Antibiotic resistance and clonal complexes with the statistically significant correlations (p <= 0.05).

**Additional file 6. **Individual accession numbers of Egyptian HA-MRSA.

## Data Availability

All raw sequence data has been deposited in the NCBI Sequence Read Archive (SRA) associated with BioProject PRJNA964454 (https://www.ncbi.nlm.nih.gov/bioproject/PRJNA964454). Individual accession numbers can be found in additional file 6.

## Abbreviations

MRSA: Methicillin-resistant *Staphylococcus aureus*
CA: Community acquired
HA: Hospital acquired
CC: Clonal complex
ST: Sequence type
AMR: Antimicrobial resistance
MGE: Mobile genetic elements
PBP: Penicillin-binding protein
MDR: Multidrug resistant
HGT: Horizontal gene transfer
PVL: Panton-Valentine leukocidin
SPA: Staphylococcal protein A
GEN: Gentamycin
ERY: Erythromycin
CD: Clindamycin
CIP: Ciprofloxacin
COT: Cotrimoxazole
C: Chloramphenicol
LZ: Linezolid
FQ: Fluoroquinolones
