# Supplementary figures and images for: Recombination-mediated dissemination of Methicillin-resistant S. aureus clonal complex 1 in the Egyptian health care settings
Source: Ann Clin Microbiol Antimicrob. 2023 Dec 14;22:109. doi: 10.1186/s12941-023-00659-y (PMC10722846; doi:10.1186/s12941-023-00659-y)

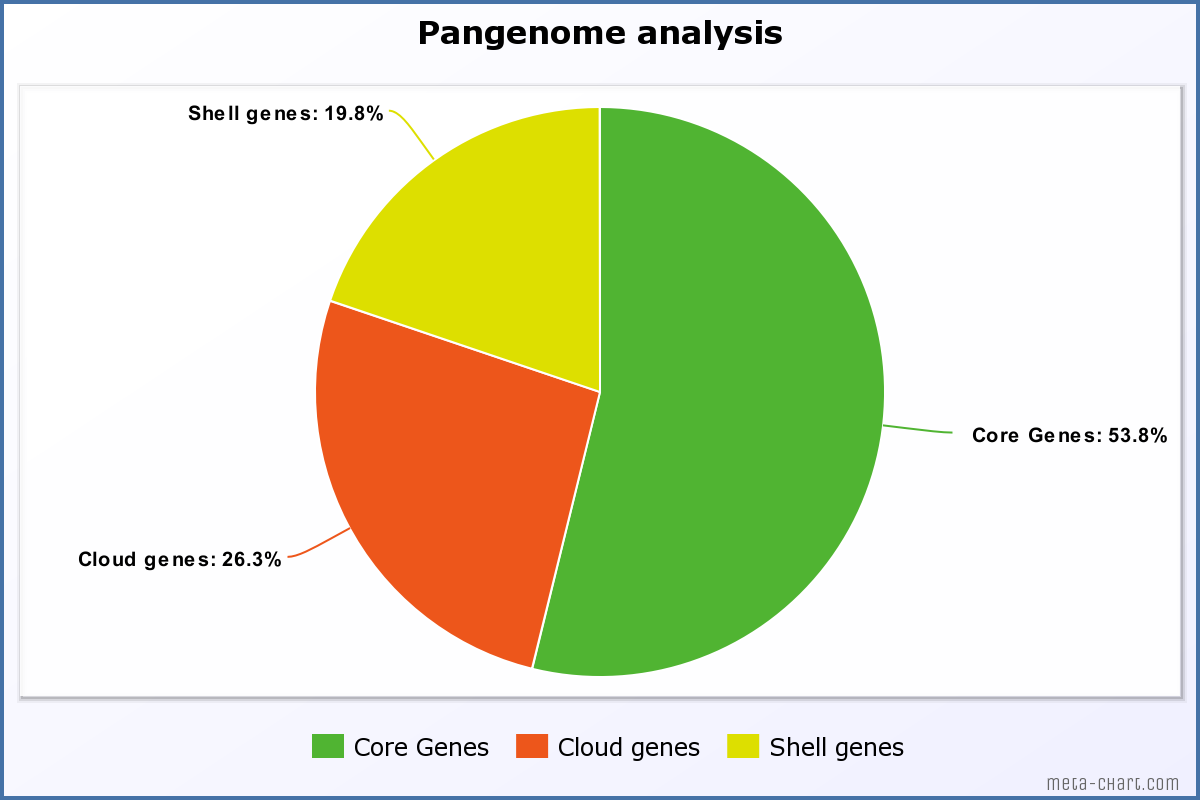

Supplement: Supplementary file 2 — Additional file 2. Pie chart for Pangenome analysis, 1204 gene were considered as core genes, 589 cloud genes, and 443 shell genes. [file 12941_2023_659_MOESM2_ESM.png]

A

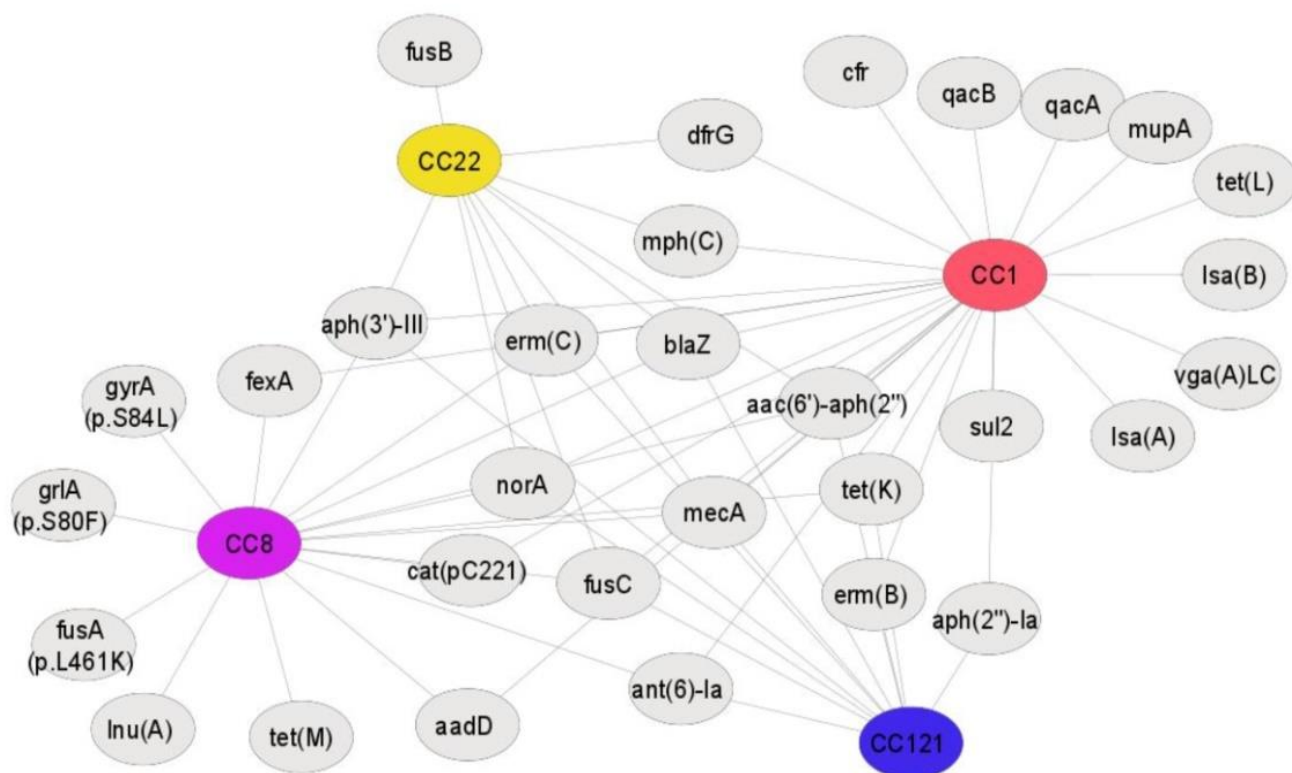

B

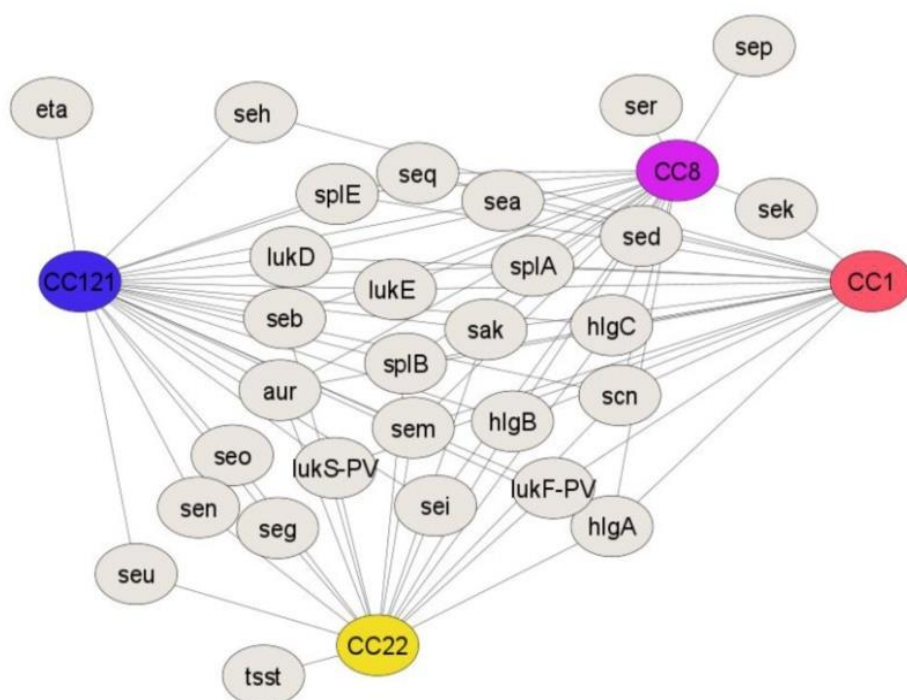

Supplement: Supplementary file 4 — Additional file 4. Distribution of antibiotic resistance profiles (A) and virulence (B) genes in the four major clonal complexes (CC1, CC121, CC8 and CC22). [file 12941_2023_659_MOESM4_ESM.pdf]

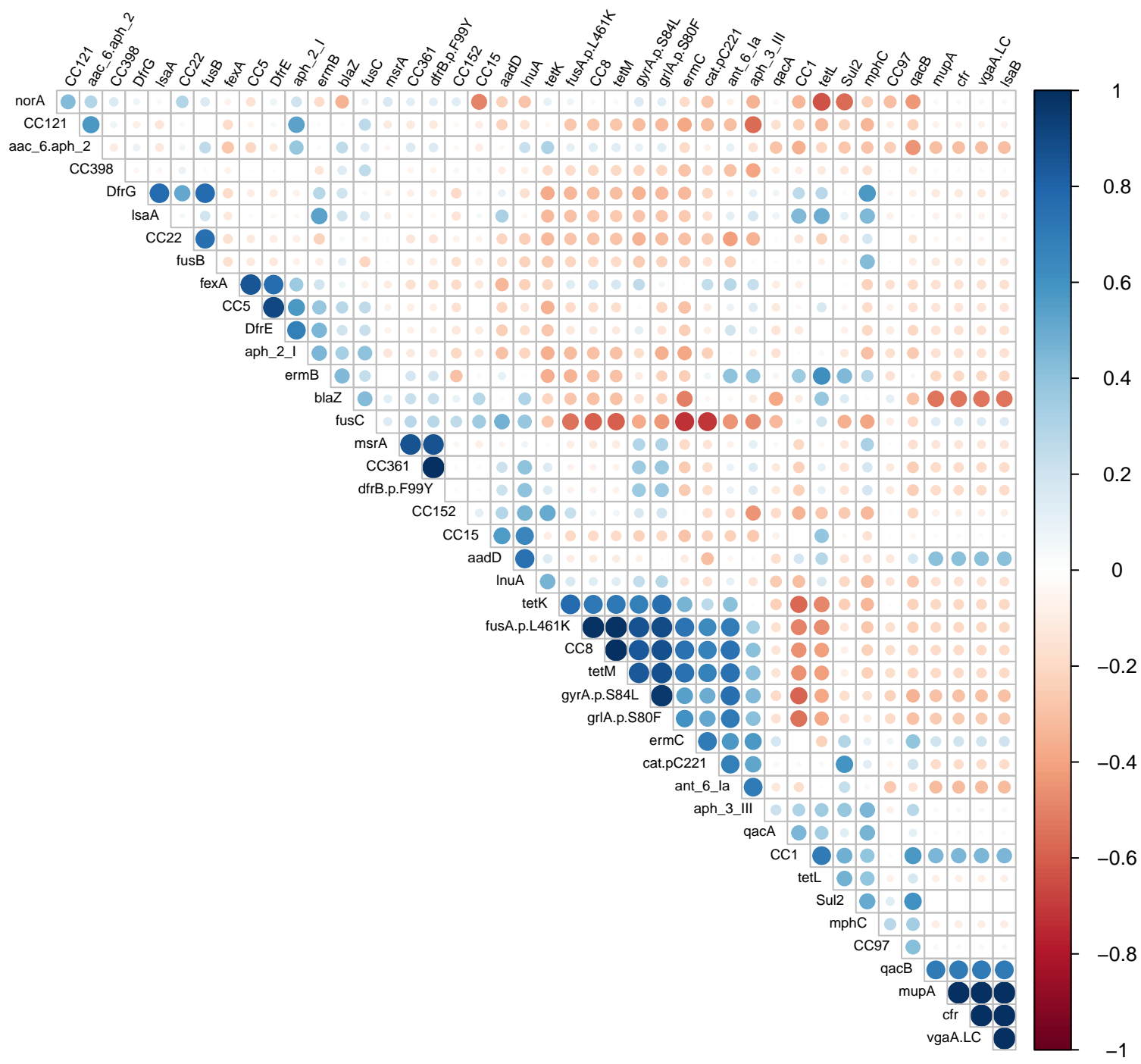

Supplement: Supplementary file 5 — Additional file 5. Correlation matrix between Antibiotic resistance and clonal complexes with the statistically significant correlations (p <= 0.05). [file 12941_2023_659_MOESM5_ESM.pdf]
